# ProNet DB: a proteome-wise database for protein surface property representations and RNA-binding profiles

**DOI:** 10.1093/database/baae012

**Published:** 2024-04-01

**Authors:** Junkang Wei, Jin Xiao, Siyuan Chen, Licheng Zong, Xin Gao, Yu Li

**Affiliations:** Department of Computer Science and Engineering (CSE), The Chinese University of Hong Kong (CUHK), Chung Chi Rd, Ma Liu Shui, Hong Kong SAR 999077, China; Department of Computer Science and Engineering (CSE), The Chinese University of Hong Kong (CUHK), Chung Chi Rd, Ma Liu Shui, Hong Kong SAR 999077, China; Computer Science Program, Computer, Electrical and Mathematical Sciences and Engineering (CEMSE) Division, King Abdullah University of Science and Technology (KAUST), Thuwal 23955, Kingdom of Saudi Arabia; KAUST Computational Bioscience Research Center (CBRC), King Abdullah University of Science and Technology, Thuwal 23955, Kingdom of Saudi Arabia; Department of Computer Science and Engineering (CSE), The Chinese University of Hong Kong (CUHK), Chung Chi Rd, Ma Liu Shui, Hong Kong SAR 999077, China; Computer Science Program, Computer, Electrical and Mathematical Sciences and Engineering (CEMSE) Division, King Abdullah University of Science and Technology (KAUST), Thuwal 23955, Kingdom of Saudi Arabia; KAUST Computational Bioscience Research Center (CBRC), King Abdullah University of Science and Technology, Thuwal 23955, Kingdom of Saudi Arabia; Department of Computer Science and Engineering (CSE), The Chinese University of Hong Kong (CUHK), Chung Chi Rd, Ma Liu Shui, Hong Kong SAR 999077, China; The CUHK Shenzhen Research Institute, 4 Gaoxin Ave Nanshan, Shenzhen 518057, China; Institute for Medical Engineering and Science, Massachusetts Institute of Technology, 45 Carleton Street, Cambridge, MA 02142, USA; Wyss Institute for Biologically Inspired Engineering, Harvard University, 201 Brookline Avenue, Boston, MA 02215, USA; Broad Institute of MIT and Harvard, Merkin Building, 415 Main Street, Cambridge, MA 02142, USA

## Abstract

The rapid growth in the number of experimental and predicted protein structures and more complicated protein structures poses a significant challenge for computational biology in leveraging structural information and accurate representation of protein surface properties. Recently, AlphaFold2 released the comprehensive proteomes of various species, and protein surface property representation plays a crucial role in protein-molecule interaction predictions, including those involving proteins, nucleic acids and compounds. Here, we proposed the first extensive database, namely ProNet DB, that integrates multiple protein surface representations and RNA-binding landscape for 326 175 protein structures. This collection encompasses the 16 model organism proteomes from the AlphaFold Protein Structure Database and experimentally validated structures from the Protein Data Bank. For each protein, ProNet DB provides access to the original protein structures along with the detailed surface property representations encompassing hydrophobicity, charge distribution and hydrogen bonding potential as well as interactive features such as the interacting face and RNA-binding sites and preferences. To facilitate an intuitive interpretation of these properties and the RNA-binding landscape, ProNet DB incorporates visualization tools like Mol* and an Online 3D Viewer, allowing for the direct observation and analysis of these representations on protein surfaces. The availability of pre-computed features enables instantaneous access for users, significantly advancing computational biology research in areas such as molecular mechanism elucidation, geometry-based drug discovery and the development of novel therapeutic approaches.

**Database URL:**  https://proj.cse.cuhk.edu.hk/aihlab/pronet/.

## Introduction

Proteins perform vital functions in a variety of cellular activities, and protein-molecule interactions decipher the complexity of organisms such as gene expression regulation (25), signal transduction (1) and drug therapy (17). However, the intricate mechanisms underlying most protein-molecule interactions remain elusive, impeding advancements in both mechanistic biology and pharmaceutical development. The interaction process between proteins and molecules fundamentally relies on the recognition of protein surfaces, where characteristics such as hydrophobicity, charge distribution, hydrogen/electron donor and binding steric hindrance. Thus, a comprehensive and efficient representation of the protein surface is essential to elucidate the mechanism of protein-molecule interaction. For example, Rudden *et al.* (21) demonstrated the utility of a single volumetric descriptor that encapsulates both electrostatic properties and local dynamics of the protein surface for protein docking, achieving a notable average success rate of 54%. Traditional experimental techniques like NMR-based measurements (2) and hydrophobic interaction chromatography (16) for assessing protein surface properties can be labor intensive and expensive. Furthermore, with the advent of the AlphaFold2 Protein Structure Database (23), a vast array of protein structures have been computationally predicted, signaling that conventional methodologies may not be sufficient to address the rapid evaluation of protein surface properties in this burgeoning dataset.

To circumvent the constraints of experimental methods, a variety of *in silico* techniques for analyzing protein surface properties have emerged, including MaSIF (6), FEATURE (11) and AutoDock (13). For example, AutoDock (13) assesses the biochemical properties on an atom-by-atom basis, while FEATURE (11) constructs a spatial depiction of protein atoms using concentric shells within a 7.5 Å radius from a grid point, encompassing 80 different physicochemical properties. MaSIF (6) goes further by integrating geometric attributes, such as shape index and curvature dependent on the distance with chemical properties like hydropathy, continuum electrostatics and the availability of free electrons/protons, across a geodesic radius of either 9 Å or 12 Å. Despite these tools offering great potential for downstream applications, they typically require a complex setup and are time-consuming to run. This results in inefficiency, as multiple users might redundantly compute the same protein properties locally. Theoretically, given a fixed protein structure, the same tool should yield identical surface representations. To address this issue, we have developed a database that pre-calculates and provides the protein surface’s physicochemical properties, such as hydrophobicity, charge distribution, potential for hydrogen bonding and interacting surfaces. These properties are encoded for protein structures obtained from both the experimentally validated Protein Data Bank (PDB) and the *in silico* AlphaFold Database (AlphaFold DB), enabling users to readily apply these features in their research. In addition, the successful *de novo* design of protein with learned surface fingerprints underscores the vital role of precise surface characterization in functionally oriented protein engineering and lays the groundwork for advancements in synthetic biology (7).

Similar to the physicochemical property, the RNA-binding landscape constitutes a crucial aspect of a protein’s surface. The ability to map RNA motifs directly onto RNA-binding proteins (RBPs) provides valuable insights into protein–nucleic acid interactions (24). A notable example is the Pumilio/FBF protein family, which modulates translation by directly recognizing specific RNA motifs, like the UGUR sequences present in RNA transcripts (20). Thus, delineating the RNA-binding profiles of RBPs is essential for a comprehensive understanding of protein-molecule interactions. In this study, we employed the state-of-the-art deep-learning framework NucleicNet (15) to predict the binding preference of RNA constituents and the binding sites on the protein surface to provide RNA-binding landscape of the protein structure from the experimentally validated database (PDB) and *in silico* database (AlphaFold DB). While our dataset is predictive, it stands out as a pioneering resource designed for immediate application in a range of fields, from enhancing Clustered Regularly Interspaced Short Palindromic Repeats and CRISPR-associated protein 9 (CRISPR/Cas) system efficiency (22) to discovering RBP-targeting therapies (10) and developing aptamer-based drug delivery systems (3).

In summary, we have developed ProNet DB, a comprehensive database dedicated to detailing protein surface features. This extensive resource encompasses physicochemical representations and RNA-binding landscapes for over 326 175 protein structures, including those from 16 model organisms within the AlphaFold DB and PDB. For each protein structure within our database, we provide not only the original molecular configuration but also a suite of surface property representations–such as hydrophobicity, charge distribution, potential for hydrogen bonding and interacting interfaces—alongside detailed RNA-binding landscapes that include sites and preferences for RNA interaction. To enable users to intuitively explore and interpret these complex surface properties and RNA-binding profiles, ProNet DB is integrated with visualization tools such as Mol* and an Online 3D Viewer. These platforms allow for the interactive and three-dimensional visualization of our comprehensive surface feature representations directly on the protein models. The server now can be assessed at https://proj.cse.cuhk.edu.hk/aihlab/pronet/, and future releases will expand the species and property coverage.

## Materials and methods

### Data source

To establish a robust foundation for ProNet DB, we began by aggregating protein structures for two key proteomes. We first collected 23 391 protein structures on *Homo sapiens* proteome and 6042 protein structures on *Saccharomyces **cerev**isiae* proteome from AlphaFold DB (23). If the corresponding experimentally validated protein structures exist in PDB, we supplemented the protein structure with the highest resolution from PDB (*H. sapiens*: 6030, *S. cerevisiae*: 1160) (4). Our pursuit of a more exhaustive database led us to further incorporate proteomes from an additional 14 model organisms, sourced from AlphaFold DB. These organisms span a diverse array of species, including plants like *Arabidopsis thaliana* and *Zea mays*, animals such as *Caenorhabditis elegans*, *Danio rerio*, *Drosophila melanogaster*, *Mus musculus* and *Rattus norvegicus*, as well as unicellular organisms including *Candida albicans*, *Dictyostelium discoideum*, *Escherichia coli*, *Glycine max*, *Methanocaldococcus jannaschii*, *Oryza sativa* and *Schizosaccharomyces pombe*. Finally, the proteomes of these model organisms sufficiently expanded ProNet DB protein structure coverage from 29 433 to 326 175 (Table 1) and led to a more comprehensive and user-friendly database.

**Table 1.** The model organism proteomes in ProNet DB

ID	Species	Name	Reference proteome	AlphaFold DB	PDB
1	*Arabidopsis thaliana*	*Arabidopsis*	UP000006548	27 434	
2	*Caenorhabditis elegans*	Nematode worm	UP000001940	19 694	
3	*Candida albicans*	*C. albicans*	UP000000559	5974	
4	*Danio rerio*	Zebrafish	UP000000437	24664	
5	*Dictyostelium discoideum*	*Dictyostelium*	UP000002195	12 622	
6	*Drosophila melanogaster*	Fruit fly	UP000000803	13 458	
7	*Escherichia coli*	*E. coli*	UP000000625	4363	
8	*Glycine max*	Soybean	UP000008827	55 799	
9	*Homo sapiens*	Human	UP000005640	23 391	6030
10	*Methanocaldococcus jannaschii*	*M. jannaschii*	UP000000805	1773	
11	*Mus musculus*	Mouse	UP000000589	21 615	
12	*Oryza sativa*	Asian rice	UP000059680	43 649	
13	*Rattus norvegicus*	Rat	UP000002494	21 272	
14	*Saccharomyces cerevisiae*	Budding yeast	UP000002311	6040	1160
15	*Schizosaccharomyces pombe*	Fission yeast	UP000002485	5128	
16	*Zea mays*	Maize	UP000007305	39 299	
				326 175	7190

### Protein surface physicochemical property

**Figure 1.**
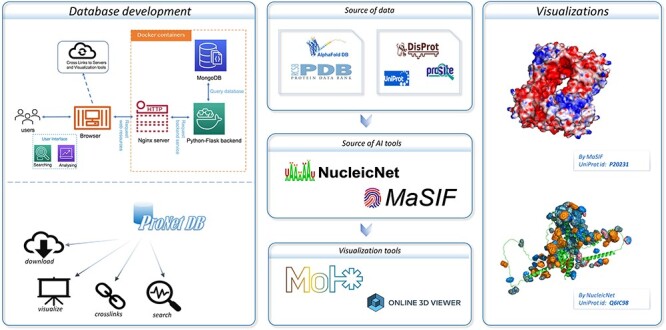
An overview of the ProNet DB and the illustration for two main outputs. The right panel shows the example of the protein surface physicochemical property and RNA-binding profiles.

The MaSIF (6) framework serves as a powerful tool for encoding protein surface fingerprints, enabling a detailed representation of a protein’s surface properties. By assigning calculated physicochemical features to each vertex of a discretized molecular surface, MaSIF provides a clear and precise depiction of the protein’s surface properties. As illustrated in Figure 1, MaSIF enables users to identify distinct regions of the protein surface, differentiating hydrophilic from hydrophobic areas and pinpointing potential interaction sites—referred to as the interacting face. We have applied the MaSIF tool to all proteins within our database, thus furnishing users with an accessible physicochemical property profile for each protein. These meticulously computed features are invaluable for a variety of downstream applications. They enhance the accuracy of binding site predictions (18), improve the predictive modeling of protein–protein interactions (9) and facilitate the innovative field of protein design (8). Indeed, recent research has underscored the importance of surface properties in function-oriented protein design, revealing that such geometric features are instrumental in advancing protein-centric research and development (7).

### Protein–RNA binding profiles

RNA–protein interactions are pivotal in a myriad of cellular processes, and understanding the dynamics between RNAs and RBPs is key to unraveling these activities. In ProNet DB, we have systematically mapped the interactions between various RNA constituents and RBPs. Utilizing the deep-learning framework established by NucleicNet (15), we have discerned both the binding preferences and the specific binding sites for multiple RNA bases across protein structures from AlphaFold DB and PDB. This includes key components such as ribose (R), phosphate (P) and the nucleobases adenine (A), guanine (G), cytosine (C) and uracil (U). The RNA-binding profiles for proteins in our database are meticulously categorized into sub-classes within each species, reflecting the diverse functional roles these proteins fulfill. ProNet DB allows users to delve into protein properties with ease, including the composition of the RNA backbone and the binding predilections for different bases. This not only paints a detailed picture of the protein–RNA binding landscape but also sheds light on broader protein surface characteristics.

## Results

### Database statistics

**Figure 2.**
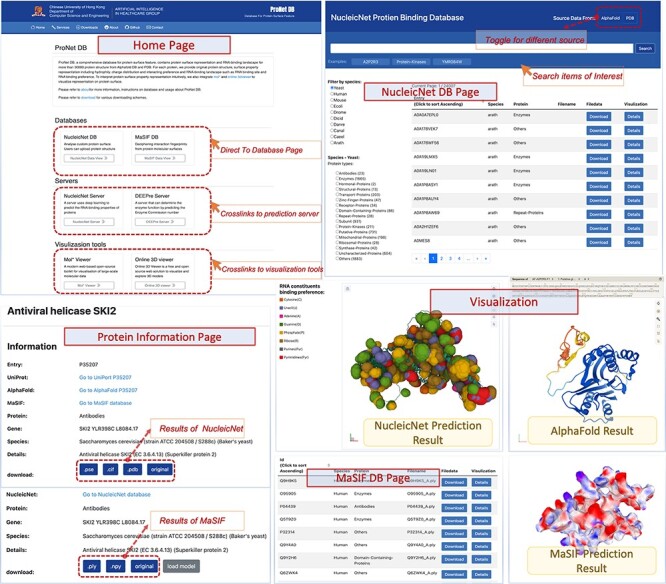
User interface of ProNet DB. Top-left: Home page contains three subsections: servers, databases and visualization tools. Top-right: NucleicNet DB page. Users can search, filter and view the searched results. On the top-right corner of NucleicNet DB page, a toggle button provides different protein sources. Bottom: Protein information page and visualization details for each item.

**Figure 3.**
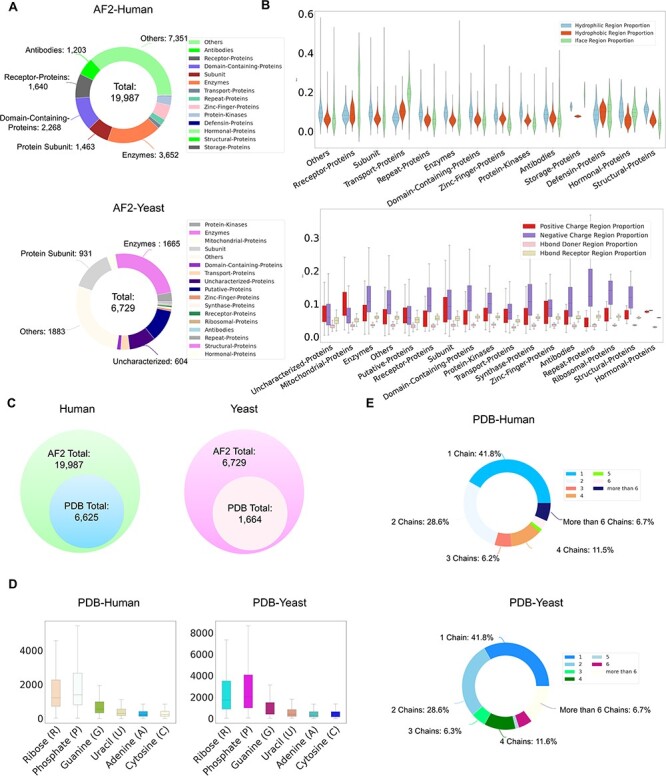
ProNet DB statistics for both human and yeast results in AlphaFold DB and PDB. (**A**) The functional classification for protein structures in both AlphaFold DB and PDB. (**B**) The upper panel illustrates the protein surface physicochemical property distribution including hydrophilic, hydrophobic and interacting face region proportion of human protein surface in AlphaFold DB. The beneath panel reveals the distribution of the positive/negative charge region and the Hbond Donor/Receptor region proportion of yeast protein surface in AlphaFold DB. (**C**) Venn diagram shows the number of experimentally validated protein structures from PDB, compared with computationally predicted structures from AlphaFold DB. (**D**) Detailed comparison of the proportion of binding profiles of each RNA constituent in PDB, e.g. four bases: adenine (A)/guanine (G)/cytosine (C)/uracil (U) and two backbone constituents: phosphate (P) and ribose (R). (**E**) The proportion of the number of chains in the PDB database in human and yeast.

Currently, ProNet DB encompasses an expansive collection of proteome entries, spanning over 16 model organism species and totaling 333 365 records from both AlphaFold DB and PDB. Our web interface, depicted in Figure 2, serves as the gateway to the database and is carefully segmented into three primary sections: prediction tools, database queries and visualization tools. The entries are detailed as follows: *H. sapiens* accounts for 23 391 from AlphaFold and 6030 from PDB, while *S. cerevisiae* contributes 6040 and 1160, respectively, in addition to the contributions from 14 other model species (Table S1). We have organized these proteins into functional sub-classes, including categories such as antibodies and enzymes. For *H. sapiens*, the protein structures are distributed among 15 sub-classes: antibodies, contractile proteins, enzymes, hormonal proteins, structural proteins, storage proteins, transport proteins, zinc-finger proteins, receptor proteins, domain-containing proteins, defensin proteins, repeat proteins, subunit, protein kinases and an others category for unclassified proteins. From Figure 3A, we observe that despite a significant number of structures being labeled as unknown (7351 entries), the majority of human protein types are clustered among enzymes (3652 entries), domain-containing proteins (2268 entries) and receptor proteins (1640 entries). In Figure 3B, we present a comparison between the proportions of hydrophobic and hydrophilic vertices against the interacting face proportion for AlphaFold2 Human proteins. A notable pattern shows that the hydrogen bond (H-bond) receptor region is statistically more significant than the H-bond donor region in AlphaFold Yeast proteins. Furthermore, Figure 3C indicates that a substantial proportion of protein structures remain unverified by experimental methods, with 66.9% for *H. sapiens* and 75.2% for *S. cerevisiae*. The accuracy of protein structure predictions is highlighted in Supplementary Figure S2, where 80.6% of validated *H. sapiens* proteins and 74.8% of validated *S. cerevisiae* proteins exhibit an RMSD ≤ 2.0. In addition, Figure 3D shows that *S. cerevisiae* proteins have a higher overall number of nucleic acids compared to those of humans. The statistical distribution of chain numbers in both PDB human and yeast datasets is depicted in Figure 3E. For a more comprehensive statistical analysis of human and yeast data, please refer to Supplementary Figure S1, while information on the proteomes of other model organisms is available in Supplementary Table S1.

### Case study

Here, we have utilized the CRISPR/Cas9 gene editing system as a case study to investigate protein–nucleic acid interactions, a topic of considerable interest due to the system’s widespread use and potential for precise genetic modifications. The Cas9 protein plays an important role in the CRISPR/Cas system, and thus, understanding how Cas9 mediates RNA-guided DNA recognition is an essential part of improving the gene editing system. The crystal structure of *S. aureus* Cas9 (PDB: 5AXW) was chosen for protein surface physicochemical property and RNA-binding profile analysis. This in-depth analysis is crucial for enhancing our understanding of Cas9’s mechanism of action and for the ongoing refinement of the CRISPR gene editing technology.

**Figure 4.**
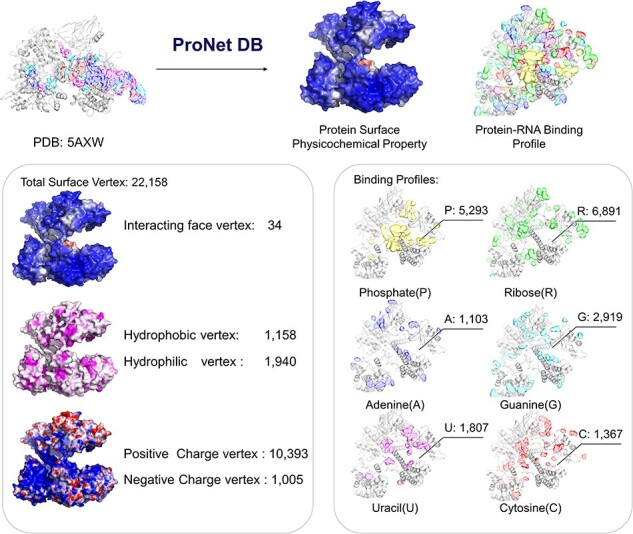
Case study (PDB: 5AXW): ProNet DB shows comprehensive information of the protein structure surface fingerprints as well as the protein–RNA binding landscape. On the left panel: Iface region is consistent with the nucleic acid-binding sites, and electron donor region is located at non-binding sites. On the right panel: The RNA-binding landscape shows the RNA-binding sites located at the inner region.

As shown in Figure 4, we have highlighted the SaCas9–single guide RNA (sgRNA) chimeric complex structure with its binding guide RNA, in which a central channel was formed in the middle of the structure. ProNet DB’s analysis of the protein surface fingerprint, particularly within the Iface region, reveals the nucleic acid-binding site’s prominence on the interacting face as opposed to non-binding areas. Moreover, the electron donor region demonstrates a positive charge within the inner area, suggesting a strong interaction between the protein surface and nucleic acids in the central channel, which spans between the recognition and nuclease lobes (14, 19). In Figure 4, our predictions of protein–RNA binding profiles, inclusive of specific RNA-binding sites and preferences, corroborate the physical presence of the RNA molecule within the inner confines of the protein structure, aligning with experimental evidence. These findings affirm that our *in silico* methodology is adept at capturing the intricate physicochemical properties of the protein surface and the RNA-binding landscape. This paves the way for future applications such as the design of sgRNAs (5) and the enhancement of CRISPR system functionality (12).

## Code availability

The ProNet DB has conducted multi-scale data analyses on a vast array of entries, encompassing 326,175 proteins across 16 different model organism species, sorted into a multitude of functional categories. The homepage (Figure 2) integrates all the server tools and is divided into three major components: prediction tools, database queries, and visualization tools. An overview and interactive table present information ranging from protein name, PDB ID, UniProt ID, protein type, interacting face proportion, Hbond region proportion, positive/negative charge region proportion, and protein-RNA-binding profiles (see Table 2). This search functionality is conveniently located in the top-right section of Figure 2, enabling users to quickly and efficiently pinpoint the information they require. The protein information page provides detailed information, download link of processed protein surface feature and visualization and more information can be found on https://proj.cse.cuhk.edu.hk/aihlab/pronet/#/services. The process code is available at https://github.com/jxmelody/PronetProcess. The ProNet DB link is https://proj.cse.cuhk.edu.hk/aihlab/pronet/#/Home. All primary data are uploaded to Figshare https://figshare.com/s/83bc43fac5aec6d1e0e6.

**Table 2.** An example entry in ProNet DB shows the data content organization of one protein *17-beta-hydroxysteroid dehydrogenase type 1*

Description		Example
Basic profile	Entry ID	P14061
	Protein name	17-beta-hydroxysteroid dehydrogenase type 1
	PDB ID	1A27
	Uniprot ID	P14061
	Sequence length	327
	Gene names	HSD17B1, E17KSR, EDH17B1, EDH17B2, EDHB17, SDR28C1
	Protein type	Enzymes
	Species	*H. sapiens* (human)
	EC number	1.1, 1.1
	Hits for all PROSITE motifs	PS00061
	Disprot ID	DP00023
MaSIF profile	Number of total surface vertex	7265
	Number of Interacting face vertex	1061
	Interacting face region proportion	0.146
	Number of hydrophilic vertex	933
	Hydrophilic region proportion	0.128
	Number of hydrophobic vertex	379
	Hydrophobic region proportion	0.052
	Number of Hbond donor vertex	232
	Hbond donor region proportion	0.032
	Number of Hbond receptor vertex	400
	Hbond receptor region proportion	0.055
	Number of positive charge vertex	400
	Positive charge region proportion	0.055
	Number of negative charge vertex	816
	Negative charge region proportion	0.112
NucleicNet profile	Number of ribose	882
	Number of phosphate	925
	Number of guanine	501
	Number of uracil	200
	Number of adenine	175
	Number of cytosine	188

An entry has three profiles: ‘Basic Profile’ contains basic information like the protein names, protein types, gene names, as well as the mapping id to other databases; ‘MaSIF Profile’ includes the physicochemical properties computed by MaSIF, describing the protein surface features; ‘NucleicNet Profile’ contains the RNA-binding preference information.

## Supplementary Material

baae012_Supp
