# Supplementary figures and images for: ProNet DB: a proteome-wise database for protein surface property representations and RNA-binding profiles
Source: Database (Oxford). 2024 Apr 1;2024:baae012. doi: 10.1093/database/baae012 (PMC10984565; doi:10.1093/database/baae012)

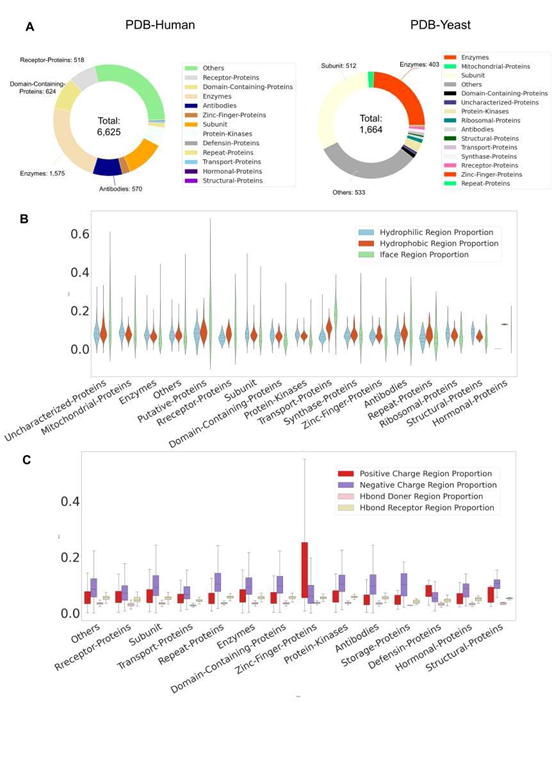

Supplement: baae012_Supp [file baae012_supp.zip › suppl_data/Figure1_Supp.png]

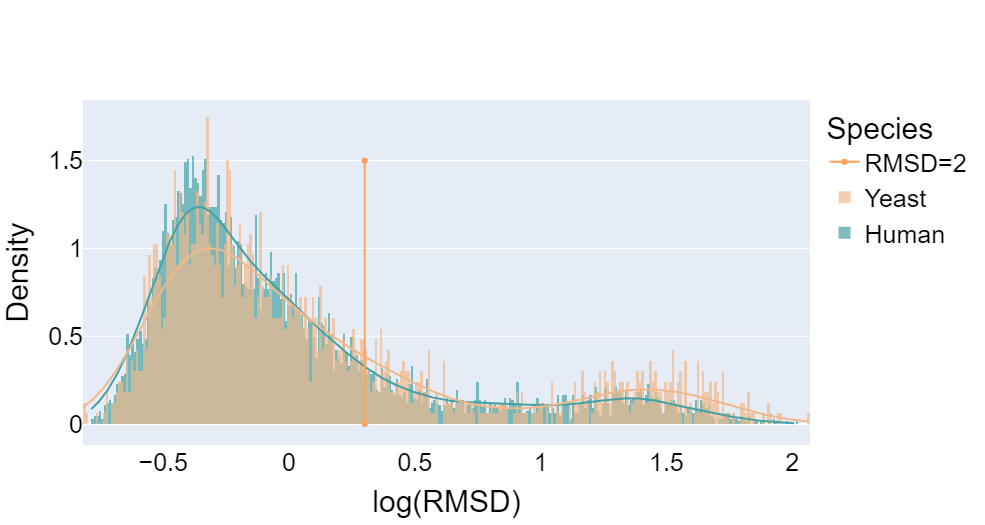

Supplement: baae012_Supp [file baae012_supp.zip › suppl_data/Figure2_Supp.png]

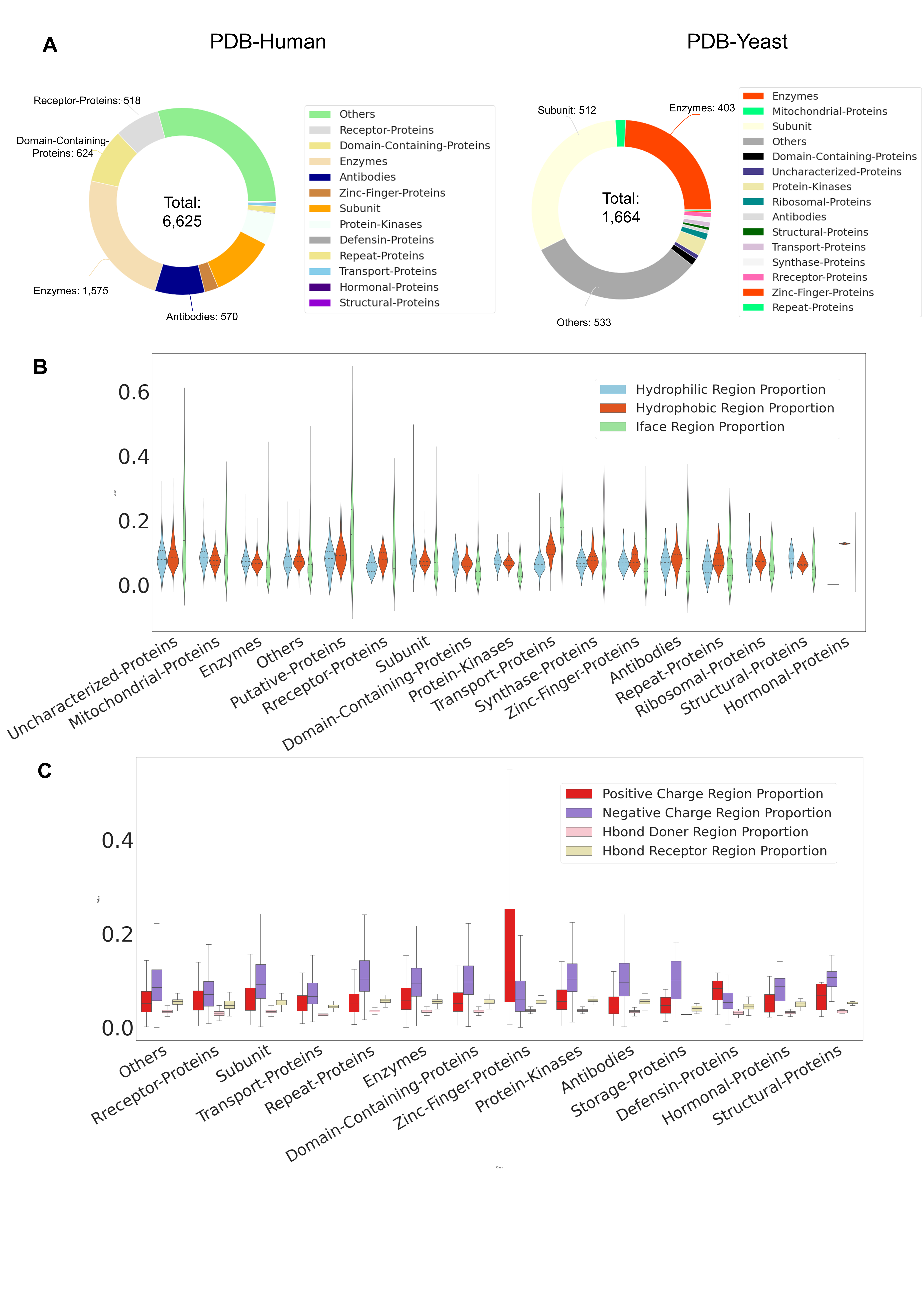

Supplement: baae012_Supp [file baae012_supp.zip › suppl_data/Figure3_Supp.png]
